# Loss of genetic diversity and inbreeding in Kashmir red deer (*Cervus elaphus hanglu*) of Dachigam National Park, Jammu & Kashmir, India

**DOI:** 10.1186/1756-0500-6-326

**Published:** 2013-08-16

**Authors:** Mukesh, Lalit K Sharma, Ved P Kumar, Samina A Charoo, Nipun Mohan, Surendra P Goyal, Sambandam Sathyakumar

**Affiliations:** 1 Wildlife Institute of India, Post Box # 18, Chandrabani, Dehradun 248 001, Uttarakhand, India; 2 Indian Council of Forestry Research and Education, Post Box # New Forest, Dehradun 248 006, Uttarakhand, India; 3 Department of Wildlife Protection, Government of Jammu & Kashmir, Srinagar 190 001, Jammu and Kashmir, India

**Keywords:** Kashmir red deer, *Cervus elaphus hanglu*, Non-invasive genotyping, Genetic diversity, Inbreeding

## Abstract

**Background:**

*Hangul* (*Cervus elaphus hanglu*), the eastern most subspecies of red deer, is now confined only to the mountains in the Kashmir region of Jammu & Kashmir State of India. It is of great conservation significance as this is the last and only hope for Asiatic survivor of the red deer species in India. Wild population of free ranging *hangul* deer inhabiting in and around Dachigam National Park was genetically assessed in order to account for constitutive genetic attributes of *hangul* population using microsatellite markers.

**Results:**

In a pool of 36 multi-locus genotypes, 30 unique individuals were identified based on six microsatellite loci. The estimated cumulative probability of identity assuming all individuals were siblings (P_ID_ sibs) was 0.009 (9 in 1000). Altogether, 49 different alleles were observed with mean (± s.e.) allelic number of 8.17 ± 1.05, ranging from 5 to 11 per locus. The observed heterozygosity ranged between 0.08 and 0.83, with mean 0.40 ± 0.11 and the inbreeding coefficient ranged between −0.04 and 0.87 with mean 0.38 ± 0.15. Majority of loci (5/6) were found to be informative (PIC value > 0.5). All loci deviated from Hardy-Weinberg equilibrium except Ca-38 (*P* > 0.05) and none of the pairs of loci showed significant linkage disequilibrium except the single pair of Ca-30 and Ca-43 (*P* < 0.05).

**Conclusions:**

The preliminary findings revealed that *hangul* population is significantly inbred and exhibited a low genetic diversity in comparison to other deer populations of the world. We suggest prioritizing the potential individuals retaining high heterozygosity for *ex situ* conservation and genetic monitoring of the *hangul* population should be initiated covering the entire distribution range to ensure the long term survival of hangul. We speculate further ignoring genetics attributes may lead to a detrimental effect which can negatively influence the reproductive fitness and survivorship of the *hangul* population in the wild.

## Background

*Hangul* or Kashmir red deer (*Cervus elaphus hanglu*), one of the four eastern most distributed subspecies of red deer found in the Indian sub-continent, is of great conservation significance since it is the only Asiatic survivor of the red deer inhabiting the broadleaf forest and temperate grassland habitats of Zanskar mountain range in Jammu and Kashmir [1]. Historically, the *hangul* was widely distributed in the mountains of Kashmir Himalayas, Chenab valley and some parts of Chamba district in Himachal Pradesh [2]. Over time, the distribution range and population size of the *hangul* deer have shrunken dramatically due to environmental and anthropogenic pressures, mainly in the form of habitat loss due to deforestation, degradation and encroachment [2,3]. The current distribution of the species is largely confined to Greater Dachigam Landscape (*ca*. 1000 km^2^) encompassing the Dachigam National Park and its adjoining protected areas [3,4]. The estimated individuals in 1900s were about 3000-5000 which had shrunk down to about 1000-2000 by 1947 and subsequently reported as low as 180-250 in 1965 [5]. The estimated individuals left over in the area till 2011 were 218 ± 13.96 [6]. *Hangul* is considered as least concern (LC) under IUCN red data list of threatened species but it is placed under Schedule-I of the Indian Wildlife (Protection) Act, 1972 and Jammu & Kashmir Wildlife (Protection) Act, 1978. It has also been listed among the top 15 conservation priority species by Government of India [7]. The Department of Wildlife Protection, Government of Jammu & Kashmir in collaboration with Wildlife Institute of India, Dehradun has been monitoring the *hangul* population by adopting scientifically robust methods since 2004. The information on the ecology of *hangul* is inadequate and only a few short terms ecological studies have been conducted majority of them dealing with habitat use, feeding habits, distribution and conservation assessment surveys [2-4,8,9]. Most importantly, the information on the genetic diversity of *hangul* population is lacking while similar studies have been conducted on other sympatric red deer species [10-12].

Genetic characterization of a species is considered necessary to formulate breeding policies and prioritize the conservation action plans in an effective and meaningful way [13,14]. Therefore, we attempted to assess the existing genetic diversity and the extent of inbreeding in *hangul* population of Dachigam National Park using the pneumatic shed hair samples collected opportunistically during the *hangul* population estimation exercise carried out in March, 2011. We took advantage of the microsatellite markers that have been cloned for Chital deer (*Cervus axis*) for analyzing genetic diversity of *hangul* population [15] since cross species amplification of microsatellites among closely related species have been used for the genetic characterization of the species for which microsatellites have not been cloned or the information is limited [16-18].

## Methods

### Sample collection and DNA isolation

**Figure 1:**
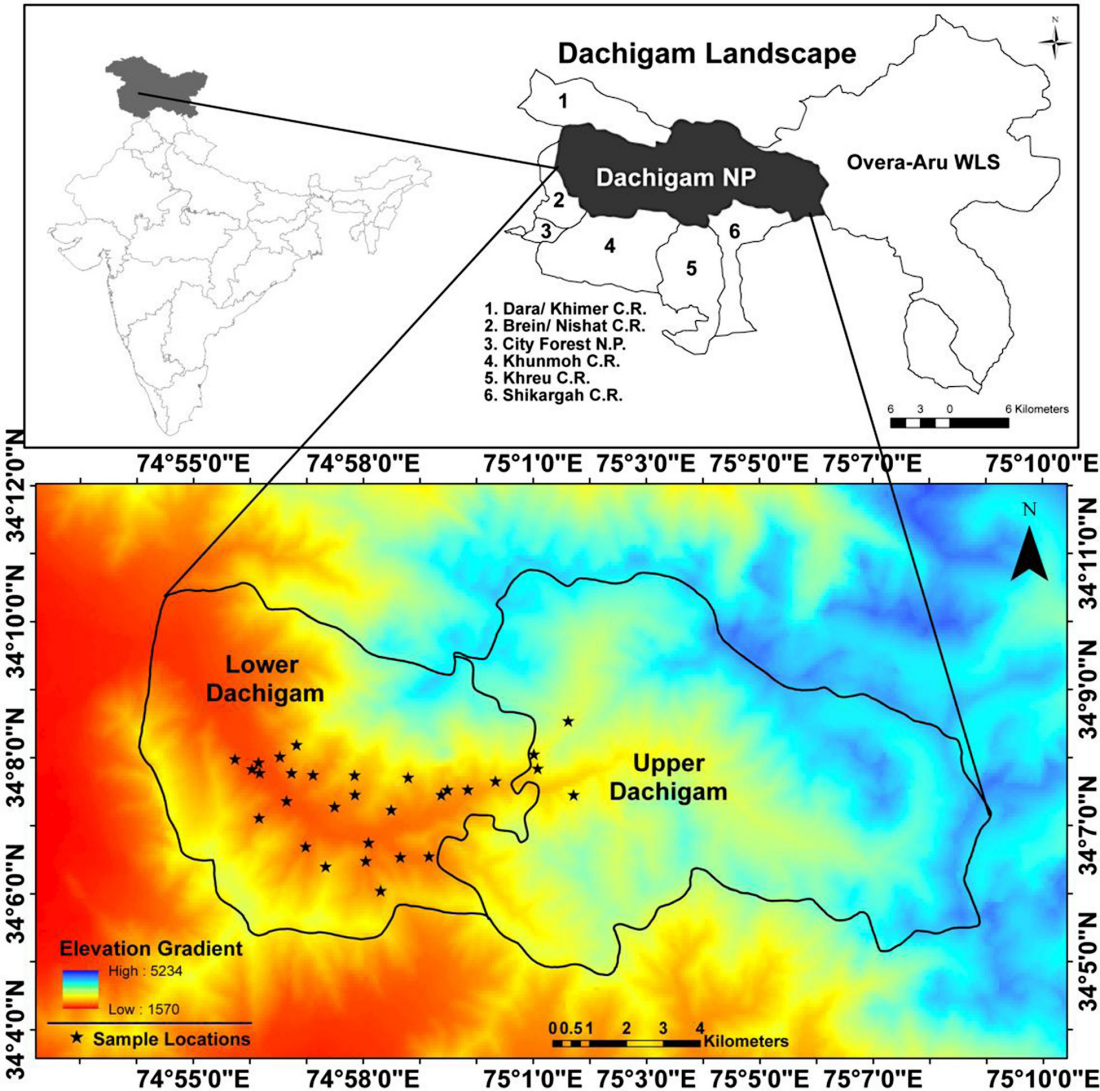
**Map showing digital elevation model of Dachigam National Park, Kashmir and its adjoining protected areas along with the sample locations of *hangul*.**

Like the other red deer species, the *hangul* also shed their pneumatic hairs during onset of spring which act as an insulator by protecting them from severe cold during winter. We participated in the *hangul* population estimation exercise carried out in March, 2011 by the Department of Wildlife Protection, Jammu & Kashmir and collected individual tuft of pneumatic hair samples (n = 84). The samples were stored in parchment paper envelope in dry condition till shipment to the laboratory for genetic analysis. The locations of the collected samples were recorded using geographical positioning system and plotted on the map using ArcGis 9.3 [19] (Figure 1). The genomic DNA was extracted using DNeasy blood and tissue kit (Qiagen, Germany) following manufacturer’s protocol with slight modification [16].

### Polymerase chain reaction and microsatellite genotyping

A set of seven microsatellites which were originally isolated from Chital deer and tested for cross amplification in several other cervids were selected for the present study [15]. The genotyping was carried out in two consecutive multiplex (Mplex) PCRs *i.e.* Mplex 1 (Ca-30, Ca-38, Ca-42 and Ca-43) and Mplex 2 (Ca-13, Ca-18 and Ca-60) using Qiagen Multiplex PCR Kit (Qiagen, Germany). PCR reactions were set up in a 15 μl of reaction volume containing 7.5 μl of 2× Qiagen Multiplex PCR Master Mix, 0.30 μl of 10 μM of each primer pair, 1 μl of Q solution (supplied with kit), 2 μl of DNA elutant (approx. 20 ng) and remaining RNase-free water to make the final reaction volume 15 μl. PCR amplification were performed on ABI 9600 Fast Thermocycler (Applied Biosystems, USA). The amplification conditions were 15 min initial heat-activation of Hot Start *Taq* DNA polymerase at 95°C, followed by 35 cycles of denaturation at 94°C for 30 s, annealing at specific temperature (Ta- 60°C for Mplex 1 and 58°C for Mplex 2) for 90 s and extension at 72°C for 60 s with a final extension at 60°C for 30 min. Fluorescence based fragment analysis was performed on ABI 3130 Genetic Analyzer (Applied Biosystems, USA) and allele scoring was done manually using GeneMapper software version 3.7 (Applied Biosystems, USA).

**Table 1:** **Genetic polymorphism of *hangul *population at Dachigam National Park, Kashmir over six microsatellite loci**

**Loci**	**Sample size**	**Allele range**	$N_{A}^{1}$	$N_{E}^{2}$	$H_{O}^{3}$	$H_{E}^{4}$	**PIC**^**5**^	$F_{\mathrm{IS}}^{6}$ **(W&C)**	$P_{\mathrm{ID}}^{7}$ **(locus)**	$P_{\mathrm{ID}}^{8}$ **(cum)**	**P**_**ID **_**sibs**^**9 **^**(locus)**	**P**_**ID **_**sibs**^**10 **^**(cum)**	$\epsilon_{1}^{11}$	$\epsilon_{1}^{12}$	$F_{\mathrm{NULL}}^{13}$
**Ca-30**	25	281-309	6	2.53	0.08	0.62	0.53	0.87	2.3E-01	2.3E-01	5.1E-01	5.1E-01	0.65	0.00	0.77
**Ca-38**†	27	163-183	7	1.68	0.44	0.41	0.38	−0.08	3.8E-01	8.8E-02	6.4E-01	3.2E-01	0.00	0.04	−0.05
**Ca-42**	30	104-138	11	4.68	0.83	0.80	0.76	−0.04	7.3E-02	6.4E-03	3.8E-01	1.2E-01	0.07	0.03	−0.04
**Ca-43**	25	225-253	11	3.78	0.44	0.75	0.72	0.42	8.7E-02	5.5E-04	4.0E-01	4.9E-02	0.11	0.00	0.26
**Ca-13**	24	205-233	9	6.26	0.42	0.86	0.82	0.51	4.4E-02	2.4E-05	3.4E-01	1.7E-02	0.00	0.00	0.33
**Ca-18**	20	126-138	5	2.09	0.20	0.53	0.47	0.63	2.8E-01	6.8E-06	5.6E-01	9.4E-03	0.14	0.08	0.43
**Mean**			8.17	3.5	0.40	0.66	0.61	0.38							
**± SE**			1.05	0.71	0.11	0.07	0.07	0.15							

$$N_{1}^{A}–$$ observed number of alleles; $N_{E}^{2}–$ effective number of alleles; $H_{O}^{3}–$ observed heterozygosity; $H_{E}^{4}–$ expected heterozygosity; PIC^5^- polymorphic information content; $F_{\mathrm{IS}}^{6}–$ inbreeding coefficient index; $P_{\mathrm{ID}}^{7}–$ (locus)- probability of identity (locus); $P_{\mathrm{ID}}^{8}–$ (cum)- probability of identity (cumulative); P_ID_ sibs^9^ (locus) - probability of identity for sibs (locus); P_ID_ sibs^10^ (cum)- probability of identity for sibs (cumulative); $\epsilon_{1}^{11}–$ allelic dropout rate; $\epsilon_{2}^{12}–$ false allele rate; $F_{\mathrm{NULL}}^{13}–$ predicted frequencies of null alleles.

† indicates no deviation from HWE (*P* > 0.05).

### Assessment of genotyping error and individual identification

Inevitable genotyping errors due to allelic drop out (ADO) and false alleles (FA) if left unaccounted, may lead to erroneous results in individual identification, population assignment, kinship and census studies [20,21]. We typed each sample twice to minimize the genotyping errors and only consensus genotypes were relied for further analysis. Maximum likelihood ADO and FA error rates were quantified using PEDANT version 1.0 involving 10,000 search steps for enumeration of per allele error rates [22]. The specific attribute of PEDANT is to estimate error rates with confidence intervals from duplicate microsatellite genotypes in the absence of reference genotypes. Unique individuals from the chunks of multi-locus genotypes were identified using GENECAP program [23]. GENECAP has the ability to include genotypes with missing scores for loci and finds matching genotypes by comparing each allele of a sample to all other alleles in all samples. The locus wise and cumulative probability of identity for unrelated individuals (P_ID_) and siblings (P_ID_ sibs) was calculated using identity analysis module in GenAlEx version 6.5 [24].

### Assessment of genetic diversity and extent of inbreeding

The per locus diversity was quantified by estimating the numbers of observed (N_a_) and effective alleles (N_e_), observed (H_o_) and expected (H_e_) heterozygosity using POPGENE version 1.32 software [25]. The polymorphic information content (PIC), an indicator of marker’s informativeness and predicted null allele frequencies were calculated using CERVUS version 3.0 [26]. For the Hardy-Weinberg equilibrium estimation, we followed the probability test approach [27] using the program GENEPOP version 4.2 [28]. The unbiased estimator of Wright’s inbreeding coefficient *F*_IS_ was calculated according to Weir and Cockerham (W&C) [29] using GENEPOP version 4.2. Linkage disequilibrium (LD) was tested using GENEPOP version 4.2 to determine the extent of distortion from independent segregation of loci following 10,000 dememorizations, 500 batches and 10,000 iterations per batch [28].

## Results and discussion

Of the total samples (n = 84) collected, 35 samples could not be used for the genetic analysis as these hair samples were either devoid of roots or too few in numbers to go for DNA extraction. Seven samples did not amplify while six samples did not produce consensus genotypes after duplicate genotyping. We observed fuzzy profile for locus Ca-60 even after repeating several times and this ambiguity was found to be consistent with all the samples forcing us to remove this locus from the initial set. Thirty six samples produced consensus genotypes after duplicate genotyping and analyzed for further analysis.

### Genotyping errors and individual identification

The observed ADO (ϵ_1_) and FA (ϵ_2_) error rates were not significant for any of the loci except for locus Ca-30 that exhibited considerably high ADO error rate *i.e.* 0.65 (Table 1). Thirty unique individuals were identified in the multi-locus genotype dataset of 36 samples. Based on six microsatellite loci, the estimated cumulative probability of identity assuming all individuals were siblings (P_ID_ sibs) was 0.009 (9 in 1000), while locus wise probability of matching genotypes among unrelated individuals (P_ID_) and siblings (P_ID_ sibs) varied from 0.30-0.04 to 0.60-0.30, respectively (Table 1). Prez *et al.*[30] observed four loci out of 31 to be polymorphic in Arabian leopards and yielded P_ID_ sibs value of 0.56-0.80. The authors established the applicability of four loci in individual discrimination where the actual population size of the area was only 10 individuals. Further, Mondol *et al.*[31] have also suggested a combination of four loci with P_ID_ sibs value of 0.03 to be applicable in discriminating among individuals from samples of common leopard collected in human dominated landscape. Mills *et al.*[32] recommended that P_ID_ less than 0.01 is necessary for studies requiring population size estimation using mark–recapture models and 0.001–0.0001 should be sufficiently low for most law enforcement forensic applications in natural populations*.* However, Waits *et al.*[33] supported to use a threshold P_ID_ sibs value double than its census size in a given area. Thus, our findings *i.e.* P_ID_ sibs 0.009 with a combination of six microsatellites indicated sufficient discriminating power to ascertain individuals for population estimation as compared to the previous studies.

### Microsatellites polymorphism and genetic diversity

All six microsatellite markers were found to be polymorphic and estimates of genetic diversity are presented in Table 1 (allelic data can be obtained upon request). Altogether, 49 different alleles were observed over 30 unique individuals with mean (± s.e.) allelic number of 8.17 ± 1.05, ranging from 5 to 11 per locus. The observed number of alleles exceeded the effective number of alleles for all the six loci. The PIC values for all six loci except Ca-38 were found to be higher than 0.5 and therefore 5/6 loci were found to be informative. Three loci *i.e.* Ca-43, Ca-13 and Ca-18 showed lower to moderate proportions of null allele frequencies while two loci did not exhibit any signature for the presence of null allele (a negative F_NULL_ values for Ca-38 and Ca-42 imply an excess of observed heterozygote genotypes). The observed heterozygosity in the population ranged between 0.08 and 0.83, with mean 0.40 ± 0.11 and expected heterozygosity ranged between 0.41 and 0.86 with mean 0.66 ± 0.07. Thus the population showed a low genetic diversity when compared to the other cervids - reindeer (0.733), red deer (0.554), roe deer (0.465; Poetsch *et al.*[34]), Chital deer (0.58; Gaur *et al.*[15]), Vietnamese sika deer (0.40-0.70; The ‘venon *et al.*[35]), musk deer (0.548-0.569; Guan *et al.*[36]). The *F*_IS_ estimates for all the six loci except Ca-38 and Ca-42 were found to be positive and therefore majority of loci (4/6) indicated heterozygote deficiency due to inbreeding with mean inbreeding coefficient to be 0.38 ± 0.15. This estimate was considerably higher than the reported estimates in other cervids - Vietnamese sika deer (0.11 to 0.27; The ‘venon *et al.*[35]) and musk deer (0.317-0.357; Guan *et al.*[36]. However, the inbreeding estimates should be explained with caution as the microsatellites showing high proportion of null alleles may influence the overall inbreeding estimates and associated parameters [37]. We compared genetic diversity estimates of *hangul* with Chital deer [15] (all six microsatellites were adopted from this study) and observed *hangul* population to display a large number of alleles with a wider size range on either side for all the six microsatellites. Such allelic range variation in the microsatellites is caused by the both contraction and expansion repeat motifs among different species [38]. In the present study, all loci except Ca-38 deviated to Hardy–Weinberg equilibrium (HWE) (*P* < 0.05) and we postulate heterozygote deficit and presence of null alleles could be the reason of HWE departure [39]. Mutations in one or both primer binding sites are the most often causes of occurrence of null alleles. This problem is particularly common while transferring microsatellites from one species to another using the same set of primers. None of the pairs of loci showed significant linkage disequilibrium except the pair of Ca-30 and Ca-43 (*P* < 0.05). Heterozygosity of locus Ca-30 was found to be offensive and the extremely low magnitude of observed heterozygosity ‘close to zero’ was duly supported by heterozygote deficiency due to large allele drop out and the presence of considerably high frequency of null allele (Table 1). We also recorded the fixation of one of the two alleles in majority of the samples and therefore present our concern to use this locus for population genetic analysis with caution. However, the locus may behave differently in other sympatric deer species as reported by Gaur *et al.*[15].

Higher observed than expected heterozygosity for loci Ca-38 and Ca-42 remained cryptic and this may be attributed with the facts that the contribution of individual breeding animals is not equal to the next generation, *e.g.* dominant males. In polygynous species such as *hangul*, the dominant males have their specific territories leading to meta-populations which certainly show allelic frequency shift over generations. Logically, a single dominant male may easily increase a particular set of alleles among his progenies as it will be preferred for mating by many of the female *hangul* deer in harems. Bebié and McElligott [40] also produces the evidence that the elevated aggression rate among female red deer in harems during oestrous, is due to the competition for selection of dominant male mates. Meaning thereby, a dominant male may have high probability to mate with different females in harems and may propagate his alleles to the subsequent generations. However, we do not refuse the possibility of stochastic or selection effects of being the elevated observed heterozygosity estimates for loci Ca-38 and Ca-42. For instance, a microsatellite locus may be near to (and so in linkage disequilibrium with) a locus under selection and hitchhiking effect can alter what we observed at these two microsatellites [41,42]. The most possible argument of ruling-out hitchhiking effect that most selections act to reduce diversity was not evidenced in our study.

## Conclusions

This preliminary study showed that the *hangul* population is considerably inbred and has a low genetic diversity when compared to other deer populations of the world. In particular, such losses of genetic diversity, reduced viability and fecundity due to inbreeding (inbreeding depression) are of concern to the long term survival of the species. We remained conservative in making strong inferences due to small sample size and involving only a few number of microsatellites. However, we present the constitutive genetic attributes of *hangul* population of Dachigam National Park that needs to be followed by the inclusion of more microsatellites for population monitoring of *hangul* in this area. We recommend the entire *hangul* population in and around Dachigam National Park should be investigated further in combination with a good sampling strategy to investigate species biology (including patterns of genetic diversity, relatedness and population connectivity) and to prioritize the potential individuals retaining high heterozygosity for conservation breeding program.

## Competing interests

The authors declare that they have no competing interests.

## Authors’ contributions

Mukesh conceived the idea, participated in its design and coordinated in writing the manuscript. LKS and SAC collected samples. Mukesh carried out PCR assays, genotyping, data analysis and wrote the manuscript. SS, SPG, LKS and SAC assisted in improving the MS with their comments. VPK and NM assisted in wet lab experiments. All authors read and approved the final manuscript.

## Authors’ information

Authors’ biography

Mukesh (Ph.D.) - Young Scientist affiliate at Wildlife Institute of India (WII), Dehradun and engaged in the conservation genetics of Cheer Pheasant in Himachal Pradesh, India. Lalit K. Sharma (Ph.D.) - Biodiversity Communication Expert at Indian Council of Forestry Research and Education (ICFRE), Dehradun, India. Samina A. Charoo (Ph.D.) – Research Officer at Department of Wildlife Protection, Jammu & Kashmir, India. Ved P. Kumar and Nipun Mohan (M.Sc.) are research scholars at WII. S.P. Goyal (Ph.D.) and S. Sathyakumar (Ph.D.) are senior wildlife biologists at WII and involved in strengthening wildlife conservation in India for over 25 years through teaching, training and research.
